# Treatment of Neurosal Affections of the Heart

**Published:** 1878-04

**Authors:** 


Treatment of Neurosal Affections of the Heart.—Having reviewed the treatment of primary affections of the heart, and also that appropriate to the interesting class of cases denominated the secondary affections of the heart, it is now fitting that the neurosal affections of the organ should be considered. These must be divided into several sections, each of which presents its own peculiar points of interest. It is of no slight importance in practice to discriminate betwixt organic disease and functional disturbance of the heart's action. In the one case there is a serious matter to deal with, and proper treatment may do much to prolong life, while inappropriate treatment may permit a valuable life to slip away prematurely. In the case of functional disturbance there is rather a distressing condition to be relieved, sometimes, however, so distressing as to render life a burden almost intolerable. A well-marked case of this last description will be given further on in this paper. There is usually no very great difficulty in distinguishing betwixt a purely neurosal condition and one where to considerable irritability is superadded a condition of incipient muscular failure—a combination far from uncommon. A still greater difficulty is experienced when there coexists actual organic disease and a neurosal condition as well; and it becomes no easy matter always to determine exactly the proportions of each factor in the case before one. In order to illustrate clearly what is meant I may refer to the case of a gentleman who consulted me sometime ago, and who had double aortic disease, with much enlargement of his left ventricle. Here there were the signs and symptoms of actual organic disease. But in addition to these there was a general condition of irritability amounting to decided erethism, which was largely felt in the heart, and its action was readily disturbed on the least excitement. There was no very marked neurosal diathesis; but the gentleman, who was a teetotaler, indulged freely in tea, which no doubt aggravated his condition. In addition, however, to this he indulged freely in marital intercourse. When the question was put to him he seemed almost relieved in his mind, and readily admitted that such was the case, stating that he had himself observed how disturbing this had become.

Here was a case where actual organic disease was blended with a decided neurosal condition. In the management of such a case there were two factors to be considered, and to be carefully kept in view; there was the treatment appropriate to the aortic mischief, and the measures suitable to the neurosal condition engrafted upon it.

It is not necessary to say anything here about the first half of the treatment. It has been discussed in a previous paper; but it is desirable to consider the first half of the management of the case. In the first place, the actual disease, and, still more, the patient's knowledge of it, predisposed him to the consideration and observance of his own symptoms, as very naturally might be the case; so that there was a morbid mental condition in a man structurally diseased. This, of course, aggravated the neurosal symptoms, as we well know undue attention to any nervous disturbance will. Then there came the effects of undue indulgence in a certain direction. This is a very important matter in connection with affections of the heart. An increasing experience shows this very distinctly. Not only is the excitement of coition often disturbing, even to healthy persons, but the generative expenditure forms a considerable tax upon an organism no longer in perfect integrity. It is not too much to say that a great deal of functional disturbance of the heart takes its origin in marital indulgence, and in this those who have had much experience either of heart disease or affectiions of the nervous system will bear me out. This is not merely amongst young men, but is found along with middle age. Much of the ill-health and of the "not-feeling-well" complained of by men who lead a quiet life amidst the most favorable surroundings is due to too great indulgence of the generative instinct. Doubtless such indulgence is less destructive in every way than promiscuous intercourse with several persons; nevertheless, it has its consequences, and they are felt very decidedly by those who labor under affections of the heart.

In another case, also, of double aortic disease, in a young man, the same disturbing effects were felt. He was engaged to be married, and consulted me as to the propriety of matrimony; a strongly negative answer was all I felt justified in giving. In many cases of functional disturbance of tho heart, the *fons et origi mali* will be found to be undue indulgence in the marital rights, and that, too, in persons who would at first sight seem the least likely people to suffer from such a cause.

When the cases present themselves where, along with actual organic disease of the heart, there is superadded a neurosal condition, it becomes very necessary to distinguish each factor in the case, and to adapt the treatment accordingly.

Being clear about this blended condition, it may now be expedient to consider the purely neurosal maladies of the heart. They consist as said before, of feveral varieties. All, however, are characterized by the presence of certain symptoms, and the absence of others. There is not that marked effect of exertion in aggravating the symptoms which is found so largely—indeed, almost universally—in actual disease of the heart itself. There is not that clear association betwixt aggravation of the ordinary condition and times when a large and excessive quantity of excrementitious matter is present in the blood, which is found in secondary affections of the heart, and where accentuation of the aortic second sound is so marked a sign. On the other hand, there are positive symptoms, and these are of two kinds, usually seen alone, but occasionally blended. The first of these is the excited action, where the heart's impulse is strong, and the patient is conscious of the blows of the heart's apex against the thoracic walls. This constitutes what, in its more marked forms, is termed palpitation. But very commonly the excited action falls short of actual palpitation, though to palpation and to ascultation an excited action of the heart is distinct enough. When the patient is perturbed, or general excitement is present, the action of the heart becomes violent, and seems as if about to shatter off the front of the chest altogether. Such is the state in some cases of chorea, where the blows of the heart upon the thoracic parietes are very violent. Not rarely in cases where there is much excited action of the heart there are intervals when the heart seems to have stopped altogether, a state often causing great alarm. Such a condition is most commonly seen in women of middle age, and occurs most frequently in the night. Their complaint is, "I am not so afraid when the heart is beating hard, for then I know it is going; but when that stoppage comes on, and I cannot feel my heart beating at all, then I feel as if I was going to die;" and that such alarm should be felt somewhat keenly by a rather nervous lady in the still solitariness of her bedroom during the night, is not a matter for any surprise. In these cases of excited action any exertion is apt to be followed by an attack of palpitation; and in this respect the case resembles one of primary disease of the heart, but with a little care a diagnosis may be made correctly, even when both maladies are coexistent.

The other form of disturbance of the heart's action is that of irregularity, or intermittency. Here the heart beats quietly in ordinary circumstances, and the alteration of rhythm is the chief feature. Not rarely this condition is found mixed up with the excited action just described, but more frequently it is found alone. The nervous relations of cardiac intermittency have been well described by B. W. Richardson, F. R. S., to whom belongs the chief merit of having shown that intermittency is often a neurosal affection, and entirely free from any connexion with organic disease. At the same time, however, marked intermittency is found along with grave and serious disease. In many cases a distinct intermission of a beat will occur in a pulse otherwise equal and rhythmical, and when so associated is usually of no serious prognostic omen. Cases of such functional disturbance of the heart are too common to render it desirable that an illustrative case or cases should be given here. They would occupy too much of the space at my disposal.

There are other cases of a more aggravated nature, where the whole nervous system is unstrung, but where the chief complaint is of the heart. Such cases are most marked and most commonly seen in women. Instead of going over the general symptoms of such cases, I prefer to relate a typical case, the more illustrative as it demonstrates what treatment may do for these sufferers.

M. H——, aged forty, a married woman with children, was brought to me at the West London Hospital on the 4th March, 1875, by my colleague, Dr. Wiltshire, as a case that would interest me. She was a woman of good physique, and intelligent beyond the majority of women of her class in life. The most marked symptom about her was an expression of uneasiness and apprehension. She was afraid of all and everything. She was afraid to be left at home; she was afraid to go out. If a knock came to the door it would give her such a start, and bring on such an attack of palpitation, that frequently she could not get across the floor to open the door. On account of its effects, especially upon her heart, she had been obliged to give up all intercourse with her husband for some years past. She always suffered from severe headache at every catamenial period. Life she described as having become a burden to her. She was put upon a mixture of bromide of potassium and digitalis in infusion of cascarilla. To regulate the bowels, she had some pills of aloes and myrrh. There were no derangements of the generative organs. Improvements speedily commenced, and on April 13th she declared hersef much better. On May 13th she reported that she had escaped the habitual nervous headache at her last catamenia. It was now determined to try the addition of quinine; and a mixture of quinine, hydrobromic acid, and digitalis, in infusion of cascarilla, was substituted for the first mixture. On June 2d she reported herself as feeling much better. She got good rest at nights; she had scarcely any palpitation, suffering less therefrom than she had done for years; the feeling of numbness extending down the right arm and hand, with which she had been troubled, was much better. For eleven years past she had not been able to walk so well as now; in fact, she described herself as feeling quite another woman. In the latter part of June she had a child ill, and was much harassed by this; yet she had not suffered from palpitation, and had no longer occasion to sit on the stairs when half-way up, as before. No nervous headache at the preceding catamenial flow. She continued to improve in every respect, but at her catamenia in July she again suffered headache from neglecting her bowels; at the next menstrual period she attended to this, and had no headache. On September 9th she described herself as feeling quite "brave;" she could cross streets, or stay at home alone at nights without discomfort, her husband having been from home for a fortnight. On October 7th she said she felt extremely well, and that she could resume her duties as a wife without discomfort. In November she said she could go to the city, and felt "quite independent at the crossings;" and, finally, in December she reported herself as feeling quite well, and asked to be discharged. Along with this general improvement her heart has become perfectly quiet, and its tumultuous stroke was exchanged for a normal beat. The look of dread and apprehension, which she wore when first seen, gradually disappeared; and in its place there grew up a look of recovering self-reliance quite in accordance with her subjective sensation. I saw the patient on December 10th, this year, and she was quite well in every respect, except headache at the catamenial periods. During the interval she has been actively engaged in her business of dressmaker.

From its general surroundings, this case of neurosal affection of the heart was one where such rapid improvement could scarcely have been looked for; nor can such happy results be attained in every case, nor even in a large proportion, when of such long standing. Nevertheless, such a case gives great encouragement to pursue a definite line of treatment in similar cases; and much relief may be afforded even when anything like complete cure, as above, is unattainable.

In connection with irritable heart must not be forgotten the effects of tea-drinking. Amongst the out-patients of hospitals this is more especially the case than in private practice; though it is far from unknown in the latter. The active principal of tea—theine—is a powerful neurotic agent, and when indulged in to excess has a very decided action upon the cardiac ganglia, rendering the heart irritable, excited, and unrhythmical in its contractions. In such cases the withdrawal of the tea is absolutely essential to successful treatment. Looked at from a chemical point of view, the principles of coffee and of cocoa are closely allied to those of tea; and it seems difficult to understand how the symptoms produced by excessive indulgence in tea are relieved by substituting for it these other allied vegetable principles. Still clinically the fact remains. It is said that tea contains,in addition to its principle, theine, a volatile intoxicating oil; and it may be the presence of this agent which makes the difference.

Another vegetable principle exercises a decided effect upon the heart—viz., tobacco. The effect of tobacco is to render the heart's action quicker, its beat feebler, and to promote a liability to palpitation. In the Royal Infirmary of Edinburgh this form of neurosal affection of the heart is recognized and known as "smoker's heart." In many cases this condition arises from great indulgence in strong tobacco; and very frequently the substitution of a lighter form of tobacco in moderation is sufficient to afford relief, without the abandonment of the favorite habit. This form of nervous affection of the heart is not so common, however, as that produced by tea.

In other cases there exists great irritability of the heart, which is closely connected with some irritation elsewhere. Several writers, and especially Botkin, of St. Petersburg, assert the pernicious effect of co-existent irritation elsewhere upon the heart; and insist that such source of irritation be totally removed, or, where that is not practicable, relieved as far as may be. Some little time ago a well-marked instance of such source of irritation came under my notice. The gentleman was the most excitable person imaginable. His heart was going at a pace which defied any correct estimate, but as a rough statement it may be said at about 150 beats per minute, with exalted action. In this case there existed great prostatic irritation, and whenever this was very troublesome the heart's action was always worse. The patient had been at Carlsbad, Homburg, Tarasp, and elsewhere, and been under many leading practitioners at home and abroad without any benefit. He could not be induced to give a systematic line of treatment a fair trial; consequently whether it would have been successful or not cannot be positively affirmed. In such cases there would seem to be either some abnormal activity about the accelerating ganglia of the heart, or else some derangement in the inhibitory action of the pneumogastric.

A case of totally opposite character is supplied by a young gentleman now under my care. He is a tall, well-developed youth of active habits, and probably the first derangement of his heart's action took its origin in some overstrain, which has left the heart with perturbed action. He suffers from palpitation, but when seen his heart has always been beating steadily. There is, however, an occasional halt, which is distinctly felt by him, and which he describes as very unpleasant. By accident it was discovered that when set thinking the rate of his pulse became altered. I sent him on to my friend, Dr. Broadbent, who found that when talking to him about his case several intermissions took place; and on asking a question involving some thought a gradual slowing of the heart's action was produced, but no intermission. He was put upon a combination of bromide of potassium and digitalis, and has improved in every respect. He complains, however, of still feeling intermissions when thinking hard. Here it is obvious that when thinking the vagus is excited to action, and so inhibits or retards the action of the ganglia of Remak.

A distinct class of cases have been described by Da Costa, of Philadelphia, which he has denominated "irritable heart," and which are now well known. They were first noted in men serving in the severe campaings of the American civil war. The sufferers were unable to march with their comrades, had dizziness and palpitation, with pain in the chest. The pulse-rate was much affected by position, varying from 110 when up, to 80 when lying down. Such cases do not present themselves to our notice in this country in any frequency; and I can only recall one case where the symptoms of irritable heart, as given by Da Costa, were well pronounced. In this case much good arose from the administration of digitalis and bromide of potassium together.

At other times instances present themselves where there is much excitement in the heart's action combined with a generally anæmic condition. Here there is a hæmic murmur at the pulmonary orifice with palpitation at intervals. These cases are usually furnished by girls. Here digitalis and the bromide of potassium are inferior in utility to tonics, with hæmatics, and attention to the leucorrhœa, which is almost invariably present. The diagnosis of these cases is not difficult; there is the objective sign of palpitation, the hæmic murmur, the *bruit de diable*, and the obvious anæmia. The subjective symptoms are—shortness of breath, especially when going up stairs, due to the deficiency of red blood-corpuscles; palpitation; and often general nervousness with vertical headache. In such cases there there is often menorrhagia as well as leucorrhœa, and attention to these matters is absolutely essential to successful treatment. Some time ago a young lady was sent to me from Torquay with the above symptoms. Her medical attendant saw that she was pale and anæmic, and found a systolic bruit, whereupon he sent her up to town for further advice. The heart symptoms here were quite a secondary matter, and the true line of treatment to be adopted was that of attention to the general health.

Such are some of the forms of disturbance of the action of the heart met with in practice, without actual organic change being present. They may have different and varied associations; but the source of the disturbed action must doubtless be sought in the nervous arrangements of the heart itself. These are very much more complex than is ordinarily supposed. There are, first, the cardiac ganglia themselves, by which rhythmical movements can be carried on when the heart is removed from the body. Then there is, next, the controling or inhibitory action exercised by the pneumogastric. It is well known that excitation of the pneumogastric nerve will show the ventricular contractions, and, if powerful enough and sustained, arrest the contractions altogether. In animals it is found that the right vagus possesses this inhibitory action more powerfully than the left. Not only is this the case, but there are contained in the pneumogastric certain fibres which possess an accelerating action, and increase the rapidity of the heart's beat. Irritation of the medulla oblongata will cause an acceleration of the heart's beat if certain nerve tracts are uninjured.

Now, amidst these complex nervous arrangements, it is not always easy to distinguish what part of the mechanism is deranged, and concerned in the production of the symptoms complained of. In cases of suspended systole, or intermittance of the heart's stroke, probably there is some irritation in the vagus by which its arresting action is increased. On the contrary, in these common cases of excited and rapid action of the heart it is possible to speculate at will as to how far this increased action is due to impaired power in the vagus—the regulating and inhibitory power being from some cause diminished—or whether it finds its origin in some irritation which stimulates the accelerating fibres. We are not yet in a position to speak very dogmatically about such nervous derangements—with their objective symptoms, palpitation and unrhythmical action. Probably in a few years more this department of heart ailments will become as distinctly intelligible as are the other forms of diseases of this organ; at present each case forms a study of itself, and requires its own appropriate treatment. Consequently it is not possible to lay down axiomatic rules for treatment, as has been done in the preceding articles on primary and secondary affections of the heart. All that may be said is that in many cases relief, and even something more, can be attained by a scheme of medication which consists in the administration of tonics, together with sedative neurotics, as in the union of quinine and digitalis with the bromide of potassium or hydrobromic acid. In addition to these measures it is obviously necessary to place the system as far as possible at rest, both physically and psychically; and this is to be achieved largely by the avoidance of all forms of disturbance and excitement, especially of a sexual character. The demands upon the system must be reduced to a minimum. We all recognize how important it is to reduce all demand, such as is induced by exertion or effort, upon the heart when its muscular walls are affected. So in neurosal affections of this organ, it is equally desirable that all forms of nervous expenditure be economized to the utmost. In all cases where there are obvious derangements these must be attended to. A misplaced uterus must be replaced; the leucorrhœal flow, so commonly found, must be attended to; if there be any dyspepsia present it must be met by a suitable diet, and vegetable bitters with bismuth; whenever there is anæmia some of the lighter preparations of iron must be prescribed, in small doses at first, and always after food. Mental and physical quiet must be insisted upon; and still more, for successful treatment, the patient must be made clearly to understand that the physician is confident in his power to afford relief.—*London Lancet.*

